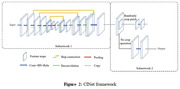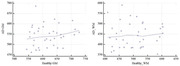# Improved gray and white matter volume loss analysis in Alzheimer's Disease using high resolution 3D MRI

**DOI:** 10.1002/alz.084171

**Published:** 2025-01-09

**Authors:** Marufjon Salokhiddinov, Dharmesh Singh, Munojat Ismailova, Dileep Kumar

**Affiliations:** ^1^ Zangiota‐2 Clinical Hospital, Tashkent Medical Academy, Tashkent, Tashkent Uzbekistan; ^2^ Central Research Institute, Shanghai, Shanghai China; ^3^ Tashkent Medical Academy, Tashkent, Tashkent Uzbekistan

## Abstract

**Background:**

Alzheimer's disease is a common neurodegenerative disease that affects the lives of millions of people worldwide. In our study, we aim to evaluate the effectiveness and accuracy of the latest automated brain volume analysis method for GM and WM analysis in health control and patients with AD and to compare optimized cut‐off values for both regions between subjects.

**Method:**

The original baseline scans from 37 HC (HC) and 39 mild AD patients downloaded from ADNI. High‐resolution T1‐weighted MRI scans were collected for each participant using a sagittal 3D MP‐RAGE sequence. Volumetric differences between groups were determined in this investigation utilizing a cascaded weakly supervised confidence integration network (CINet). Data were collected from two areas of interest (ROI): white matter volume and gray matter volume. Total CGM encompassed all the gray matter in the frontal, parietal, temporal, and occipital lobes and all gray matter that could be visually identified as being a part of the cerebral cortex. Volume was measured in cc.

**Result:**

Results showed that healthy subjects GM and WM brain volume values were significantly greater than mild AD subjects by ∼18%. The mean ± SD of GM volume for HC was 618.41 ± 50.54 cc and for mild AD patients was 542.40 ± 101.37 cc. The mean WM brain volume was 502 ± 59.60 cc for HC and 445 ± 60 cc for mild AD subjects. Both GM and WM volume values for HC showed significant (p < 0.05) higher (∼18% in both GM and WM volumes) than mild AD subjects. There was very low correlation (r = 0.15) for GM volume of HC and mild AD, and r = 0.02 for WM volume of HC and mild AD patients.

**Conclusion:**

In conclusion, the CINet combined V‐Net method has demonstrated encouraging results in quantifying GM and WM volume in MRI images of people with early‐stage AD. Compared to conventional human segmentation techniques, this automated approach has various advantages, including improved productivity, accuracy, and dependability.